# Steerable-Discrete-Cosine-Transform (SDCT): Hardware Implementation and Performance Analysis †

**DOI:** 10.3390/s20051405

**Published:** 2020-03-04

**Authors:** Riccardo Peloso, Maurizio Capra, Luigi Sole, Massimo Ruo Roch, Guido Masera, Maurizio Martina

**Affiliations:** Department of Electronics and Telecommunication (DET), Politecnico di Torino, C.so Duca degli Abruzzi 24, 10129 Turin, Italy; maurizio.capra@polito.it (M.C.); luigi.sole@studenti.polito.it (L.S.); massimo.ruoroch@polito.it (M.R.R.); guido.masera@polito.it (G.M.); maurizio.martina@polito.it (M.M.)

**Keywords:** video coding, discrete cosine transform, directional transform, VLSI

## Abstract

In the last years, the need for new efficient video compression methods grown rapidly as frame resolution has increased dramatically. The Joint Collaborative Team on Video Coding (JCT-VC) effort produced in 2013 the H.265/High Efficiency Video Coding (HEVC) standard, which represents the state of the art in video coding standards. Nevertheless, in the last years, new algorithms and techniques to improve coding efficiency have been proposed. One promising approach relies on embedding direction capabilities into the transform stage. Recently, the Steerable Discrete Cosine Transform (SDCT) has been proposed to exploit directional DCT using a basis having different orientation angles. The SDCT leads to a sparser representation, which translates to improved coding efficiency. Preliminary results show that the SDCT can be embedded into the HEVC standard, providing better compression ratios. This paper presents a hardware architecture for the SDCT, which is able to work at a frequency of 188MHz, reaching a throughput of 3.00 GSample/s. In particular, this architecture supports 8k UltraHigh Definition (UHD) (7680 × 4320) with a frame rate of 60 Hz, which is one of the best resolutions supported by HEVC.

## 1. Introduction

In recent years, high-resolution multimedia content has fostered research in the field of video compression. Indeed, in 2013 the Joint Collaborative Team on Video Coding (JCT-VC) released the High-Efficiency Video Coding (HEVC) standard, also referred to as H.265 [1].

**Figure 1:**
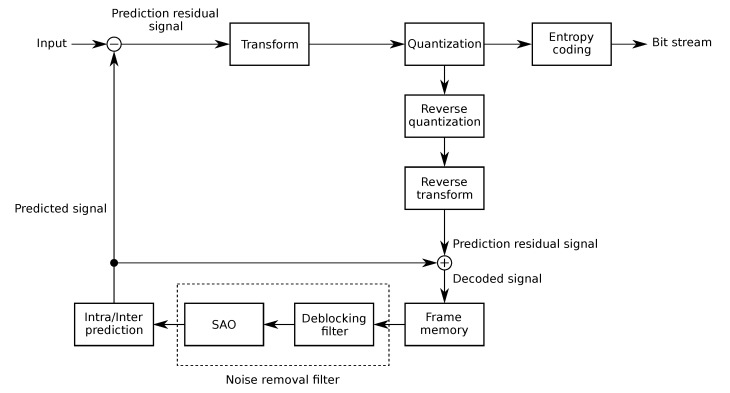
HEVC basic structure.

Interestingly, the HEVC standard improved the coding efficiency gain by reaching 50% of bit-rate reduction (for the same quality level) with respect to the previous Advanced Video Coding (AVC)/H.264 standard. Noticeably, HEVC not only improved the compression capability, but it effectivelyh andles high-quality video resolutions, enhanced frame rates, and increased dynamic range. In particular, the HEVC standard relies on coding tree units (CTUs) to improve transform coding and prediction. Each CTU contains two coding tree blocks (CTBs), one for the luma component and one for the chroma components. CTBs are partitioned into smaller blocks called coding units (CUs) along with a tree-based coding structure that includes prediction units (PUs). PUs exploit the temporal and spatial redundancies present in video streams leading to inter-frame and intra-frame prediction. The sizes of PUs vary from 8 × 4 and 4 × 8, to 64 × 64 pixels for inter-frame, while for intra-predicted PUs size goes from 4 × 4 to 32 × 32 pixels. As PUs are coded without including neighboring blocks, blocking artifacts due to discontinuous block boundaries can occur. To reduce these artifacts and to improve the quality of the decoded frames, the HEVC standard includes two in-loop filters: the deblocking filter (DBF) and the sample adaptive offset (SAO), as depicted in Figure 1.

During the prediction, for each PU, the difference between the predicted block and the current block (*residual*) is lossly coded by the means of transform and quantization. The transform stage can be either the Discrete Sine Transform (DST) or Discrete Cosine Transform (DCT). While the DST is used only for the smallest block size, namely 4 × 4 pixels, the DCT is used for all the other sizes, up to 32 × 32. For this reason, some works pointed out that the complexity of the transform stage in the HEVC context is particularly relevant [2,3]. This motivated several researchers to propose dedicated architectures for variable size DCTs, such as [4,5,6]. Recently, G. Fracastoro et al. [7] proposed the Steerable DCT (SDCT) and showed that it can give some coding advantages when embedded in the HEVC standard [8]. Such a directional transform is not tailored to any specific one, but it can be potentially applied to any two-dimensional separable transform. Moreover, it can be oriented in any selected direction, providing a more scattered representation depending on the chosen orientation. Unfortunately, such enhancements in HEVC lead to further complexity increases. These features interfere with battery-powered platforms and real-time applications, since the higher the complexity, the higher the power consumption. This current paper details the hardware accelerator for the SDCT described in [9,10]. Such an accelerator is able to support the ultimate video coding resolutions like the 8k UltraHigh Definition (7680 × 4320 pixels). After a brief introduction on the SDCT in Section 2, Section 3 analyses the proposed architecture and finally Section 4 presents the implementation results discussing possible trade-offs. Lastly, Section 5 offers an overview of the entire work by providing some results about the effectiveness of the SDCT in comparison to other canonical solutions.

## 2. Background

HEVC is a block-based video compression algorithm and, like similar compression schemes, it employs spatial transforms. In particular, the 2-D DCT is the main one, which acts along the horizontal and vertical directions. The 2-D DCT is defined as

(1) $$\begin{array}{cc} X_{k_{1},k_{2}} & =\sum_{n_{1}=0}^{N_{1}-1}\left(\sum_{n_{2}=0}^{N_{2}-1}x_{n_{1},n_{2}}\cos\left[\frac{\pi }{N_{2}}\left(n_{2}+\frac{1}{2}\right)k_{2}\right]\right)\cos\left[\frac{\pi }{N_{1}}\left(n_{1}+\frac{1}{2}\right)k_{1}\right]\\ \; & =\sum_{n_{1}=0}^{N_{1}-1}\sum_{n_{2}=0}^{N_{2}-1}x_{n_{1},n_{2}}\cos\left[\frac{\pi }{N_{1}}\left(n_{1}+\frac{1}{2}\right)k_{1}\right]\cos\left[\frac{\pi }{N_{2}}\left(n_{2}+\frac{1}{2}\right)k_{2}\right] \end{array}$$

which is, by definition, a separable transform. The DCT deals better than the DFT (Discrete Fourier Transform) with the borders of the coding blocks. This allows higher energy compaction with reduced sensitivity to quantization. It is also a real transform, thus, computations on complex numbers are not required. The operation can be stated as a convolution, leading to a compact and efficient implementation.

**Figure 2:**
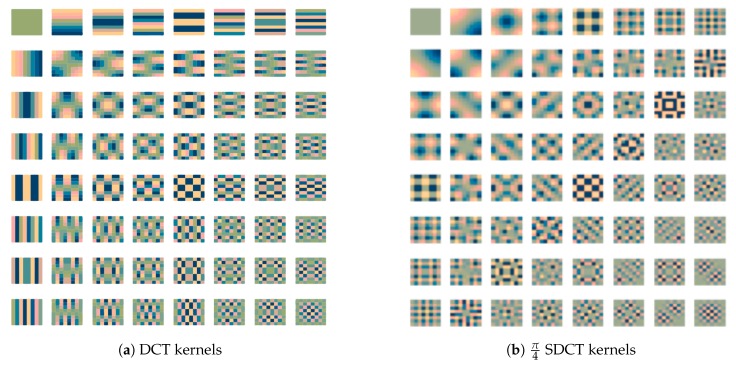
Example of Discrete Cosine Transform (DCT) and Steerable Discrete Cosine Transform (SDCT) kernels.

It is possible to demonstrate that for blocks that include diagonal edges, a directional transform will be better suited, leading to a higher compression ratio. The work of B. Zeng et J. Fu [11] presents a mathematical framework about directional DCT (DDCT). This transform is difficult to handle as it requires non-canonical DCT lengths and complex reshaping of the blocks. Recently, G. Fracastoro et al. [7] proposed the Steerable DCT (SDCT). It employs the graph Fourier transform from [12] to obtain an easier-to-handle directional DCT. The SDCT kernels still retain a square shape so that computation remains easy to perform, even though this 2-D transform is not separable in two 1-D operations as for the classic 2-D DCT. Lately, the work in [8] demonstrated that it is possible to split the steerable cosine transform into a traditional DCT followed by a geometrical rotation. The resulting kernels are the same as the SDCT but the computation workload is reduced by exploiting the 2-D DCT separability. Section 3 will better deal with this issue. Figure 2 shows different kernels obtained by the SDCT, the DCT being a special case of the SDCT with a rotation by zero degrees.

## 3. Architectural Implementation

### 3.1. Datapath

**Figure 3:**
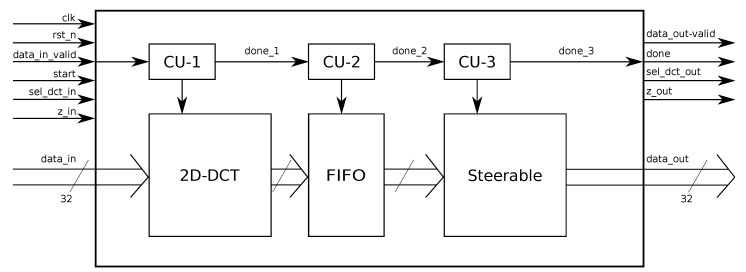
Whole SDCT structure.

While the 2D-DCT employed in HEVC is an inherently separable operation, the SDCT must be computed all at once. The complexity of a transform that is not separable is far greater than a separable one, so this may be a big drawback for the implementation. However, the complexity can be decreased drastically by splitting the SDCT into two parts, namely, a separable 2D DCT followed by some rotations, and then by computing the separable transform before applying rotations, as reported in [8]:

(2) $$\tilde{x}=T\left(\theta \right)x=R\left(\theta \right)Tx=R\left(\theta \right)\hat{x}$$

where x are the input samples, $\hat{x}$ are the results obtained by applying the *T* transform matrix, R(θ) is the rotation matrix, while $\tilde{x}$ is the result of the SDCT. Thus, the SDCT can be decomposed in a DCT followed by a steering transformation. The DCT part can be implemented as suggested in the literature using a folded architecture [13]. When all the samples returned by the 2D-DCT are available, the rotations must be applied to obtain a steering transform. Since the DCT works exploiting a sliding window approach on the data, the process takes several steps to complete. However, the results will be provided all at once. This means that the steering part of the architecture has to work faster than the DCT. This issue has been tackled in this work by defining two clock regimes, one for the 2D-DCT and one, four times faster, for the steering part, to comply with the throughput offered by the 2D-DCT transform block. A FIFO memory between the two parts acts as a buffer memory. The whole structure is depicted in Figure 3.

**Figure 4:**
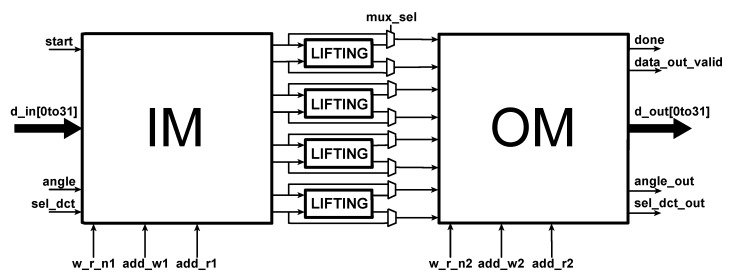
Steerable block structure.

**Figure 5:**
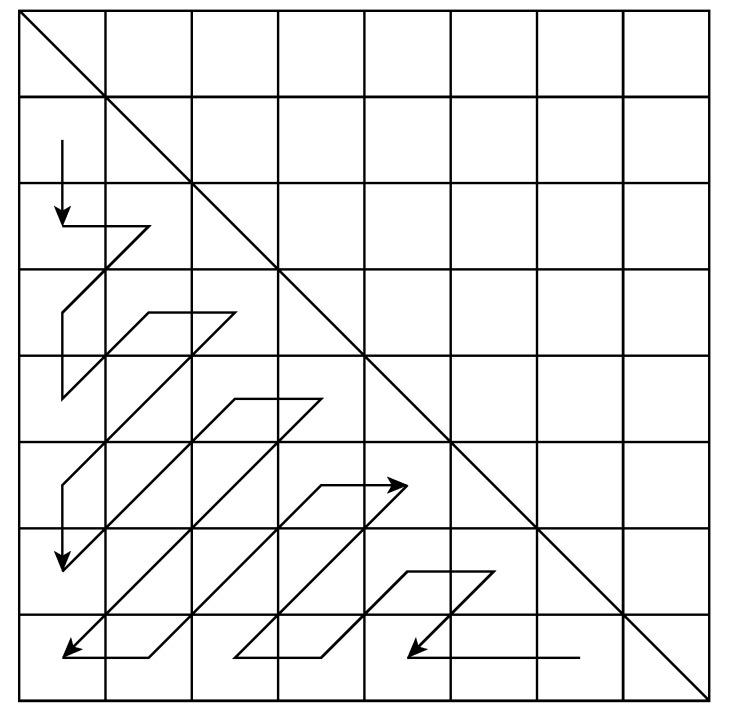
Zig-zag scanning order.

The 2D-DCT block is based on the architecture proposed in [13] by Meher et al., which is very flexible and efficient, especially when dealing with folded transforms of size 4, 8, 16 and 32. The steerable part is shown in Figure 4. It is composed of an input memory (IM), an output memory (OM) and the lifting blocks that perform the rotation [14]. Some multiplexers are used to bypass the lifting blocks for the case of no rotation, returning directly the result given by the DCT. Despite the possiblity to bypass the IM and OM blocks when no rotation has to be applied, such an alternative leads to different latency of the architecture as a function of the rotation angle. Thus, in order to simplify the interface of the architecture, we decided to only bypass the lifting blocks. The IM is required also to reorder the samples as the steering process is computed on the custom zig-zag order given in Figure 5; this is different from the classic zig-zag ordering, as the vectors are rotated in pairs with respect to the diagonal elements. Rotation by lifting scheme:

(3) $$\left(\begin{array}{cc} \cos\theta & \sin\theta \\ -\sin\theta & \cos\theta \end{array}\right)=\left(\begin{array}{cc} 1 & \frac{1-\cos\theta }{\sin\theta }\\ 0 & 1 \end{array}\right)\left(\begin{array}{cc} 1 & 0\\ -\sin\theta & 1 \end{array}\right)\left(\begin{array}{cc} 1 & \frac{1-\cos\theta }{\sin\theta }\\ 0 & 1 \end{array}\right)$$

**Figure 6:**
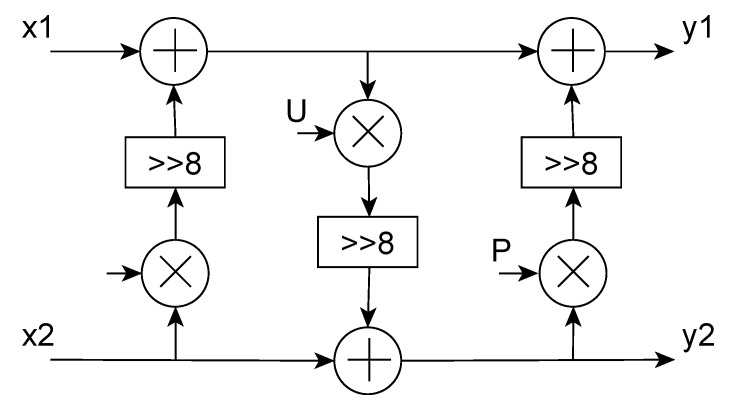
Lifting-based rotation.

The rotation matrix is decomposed in the multiplication of three other rotation matrices, in such a way that the resulting structure shown in Figure 6, presents a lower complexity. Indeed, this implementation requires only three multipliers, one less concerning the original implementation, leading to a reduction of the 25% of the computational area, shorter latency and less power consumption. To further simplify the architecture, the multiplication for P and U coefficients from Equation (3)

(4) $$P=\frac{1-\cos\theta }{\sin\theta }$$

(5) U=-sinθ

in Figure 6 are implemented as shift and add, as the number of possible rotation angles has been fixed to 8 (from 0, no rotation, to 7), as reported as optimum in [8] by M. Masera et al. The steerable block thus introduces 2×N clock cycles of latency for the reordering stage plus 4 clock cycles due to the internal pipeline. Therefore, in the event that all the SDCTs have a length N = 32, the latency is equal to 68 clock cycles, which corresponds to the worst case.

### 3.2. Control Unit

The design requires two Control Units (CUs), one for the DCT part and one for the steering part. The 2D-DCT block is managed by its control unit, which generates all the control signals and the required memory addresses. It is composed of a Finite State Machine (FSM) and a counter. The FSM is composed of two states (FWR1 and FWC1), plus an IDLE state. When the external starting input signal is received, the FSM switches from IDLE to FWR1. The counter starts to increase its value and the write_enable signal is raised so that the partial 1D-DCT results are stored in the transposition buffer at the position indicated by the counter address value. The input signal itself encapsulates the length of the current DCT and consequently the value to be reached by the counter. Once the maximum counter value (cnt_max) is reached, the FSM switches from FWR1 to FWC1. In this state, the FSM is responsible for the read memory address generation and the assertion of the data_out_valid signal. The maximum counter value in this state remains the same as the previous one. Once cnt_max is reached, the two-dimensional transformation is completed, and the FSM evolves to a new FWR1 state if the start signal is asserted again, otherwise, it returns to the IDLE state.

For what concerns the steerable block, its control unit generates all the signals needed to manage the datapath and to address the two buffers. This unit is made up of an FSM and four counters. The FSM is composed of 14 states and an IDLE state, divided into 5 functional groups. Table 1 reports all groups functionalities. All the states belonging to the same group are similar, they are distinguished only by the different behavior of the output signals and the counter threshold.

State A coincides with the start of the steering process. Here, the 2D-DCT results are written into the input buffer. After that, the FSM switches to the B state, where the data is read from the input buffer and is written to the output one after being rotated. Then the results must be removed from the output buffer. However, as the video coding application requires to process a continuous stream of data, every time the previous results are completely written in the output buffer, new values need to be fetched and stored in the input one. State C handles such a situation, allowing the architecture to provide uninterrupted input/output data flow.

In principle, these three states plus E are enough to execute the steerable operation but the execution of multiple steerable with different lengths must be considered. The FSM complexity grows with the number of different supported SDCT lengths. As stated before, this unit supports lengths of N = 4, 8, 16 and 32. Consequently, many different states are required. For instance, Table 2 shows one simple FSM execution, in which a steerable operation with length N = 16 is followed by a new operation with a length of N = 8. In this case, after the eight columns of new data are written in the input buffer, it is necessary to read and rotate them. The first N = 16 columns of the output buffer are filled with previous data, but not all of them have been read. Thus, the FSM introduces an offset in the writing address to avoid the overwrite of previous results. At this point, new data can be stored in the output buffer, while the old ones are read at the same time. In the opposite situation there are no problems: the new execution is longer than the previous one, so temporary storage is not needed.

**Figure 7:**
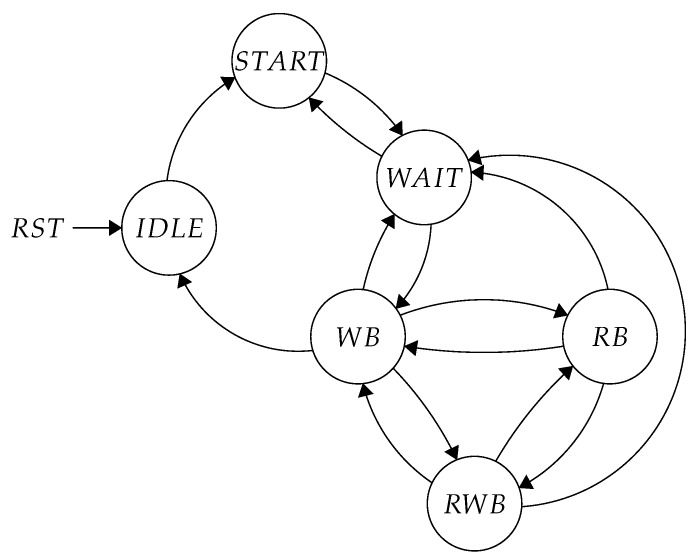
Simplified FSM diagram.

The four counters are responsible for the generation of the double buffer addresses and to control the FSM evolution from state to state. Two counters are necessary to decide the next state: while the first one takes into account the previous SDCT length, the second one deals with the current SDCT length. A third counter generates the addresses for the input buffer and the coefficients Read-Only Memory (ROM). Finally, the last counter is used to point to the SDCT results in the output memory. Figure 7 visually represents the simplified evolution of states in the control unit. The states are grouped as in Table 1:

- START: write input buffer
- WAIT: read input & write output buffer
- WB (Write Buffer): write input & read output buffer
- RWB (Read and Write Buffer): read input & write output & read output buffer
- RB (Read Buffer): read output buffer

The decision about which will be the next state depends on the the current SDCT computation phase and the size of the next SDCT to be computed, this is why the states RB, RWB and RB are so thightly interconnected.

### 3.3. Reduced SDCT Architectures

The unit presented so far can compute SDCT of lengths 4, 8, 16 and 32. This type of structure has been designed to be implemented inside the HEVC standard while providing maximum flexibility. This algorithm could be also used for video compression standards with lower constraints and image compression algorithms, such as JPEG. As these cases do not require the whole range of SDCT lengths, two reduced SDCT units have also been developed. The first can compute SDCT of length 4, 8 and 16 (called SDCT-16), while the second is capable of computing SDCT of length 4 and 8 (called SDCT-8). These two units have a reduced throughput of 50% and 75%, respectively, with a parallelism of 16 or 8 data samples instead of 32. This leads to a consistent reduction of the memory sizes. In particular, the length of both rows and columns of all memories is halved in the SDCT-16 unit, while it is four times lower in the SDCT-8 unit with respect to SDCT-32. As a result, the area occupation of these units is much lower than the SDCT-32 one, providing suitable solutions tailored to the final application. Moreover, since the throughput is reduced, just one clock domain has been used for both DCT and steerable block. In this way it is possible to remove the FIFO memory interface and lower the design complexity.

## 4. Results

In order to satisfy the HEVC stream requirements for a video resolution of 7680×4320, frame rate of 60 fps, with the YUV 4:2:0 image coding, the proposed structure needs a throughput of almost 3 GSample/s. As discussed in Section 3, the folded version presented in [13] has been chosen since this approach guarantees the required throughput. This structure has a processing rate of 16 pixels per cycle, therefore the architecture needs a frequency of at least 187 MHz ($2.99\times 10^{9}$/16 MHz). Furthermore, clock gating has been included during the synthesis process, leading to a power consumption reduction of about 58% as shown in Table 3. The technology employed for the synthesis is the UMC 65 nm. The following architectures have been considered and synthesized:

- two-dimensional DCT
- SDCT
- reduced SDCT-16
- reduced SDCT-8

Concerning the steering part, several clocks have been tested, namely 1×, 2×, 4× and 8× (with respect to the DCT clock frequency). By increasing the Steerable unit frequency it is possible to decrease the parallelism and consequently the number of input/output ports of the buffers.

It can be noticed in Table 4 that by reducing the data parallelism of the Steerable unit, the size of the input memory (IM) and output memory (OM) decreases considerably, while the size of all the other sub-blocks slightly increases, due to the synthesizer constraints with different clock regimes.

Table 5 presents an overview of the obtained results, comparing the DCT baseline with the SDCT proposed.

As it can be noticed, the area and power results of the SDCT-16 are around 60% smaller than the complete SDCT. On the other hand, the SDCT-8 area is around 75% smaller than the SDCT-16 and 90% smaller than the complete SDCT while the throughputs are reduced respectively by 50% and 75%. Finally, comparing the DCT and the SDCT architecture we can observe that the hardware overhead to support up to N = 32 is very large. However, removing the hardware support for the steering part with N = 32 (SDCT-16), the area becomes comparable with the one of the DCT. As a consequence, this solution can be of interest to increase the rate-distortion performance [8].

### 4.1. Reduced SDCT Compression Savings

The performance of the proposed encoder with a DCT directional transform is analyzed using the metric gauge Bjķntegaard Delta Bit-Rate (BDBR) [15], using the original HEVC encoder HEVC test Mode (HM-16.6) as the reference method. The full SDCT requires on average 22% more time to be executed with respect to plain DCT on an modified HM version, while SDCT-16 took on average 18% more time and SDCT-8 only 15% more time. By further optimization this overhead could be reduced to make the execution times closer to the DCT case. On one hand, negative values of BDBR stand for bit-rate savings, thus improved coding efficiency, while, on the other hand, positive values denote loss of rate-distortion.

**Figure 8:**
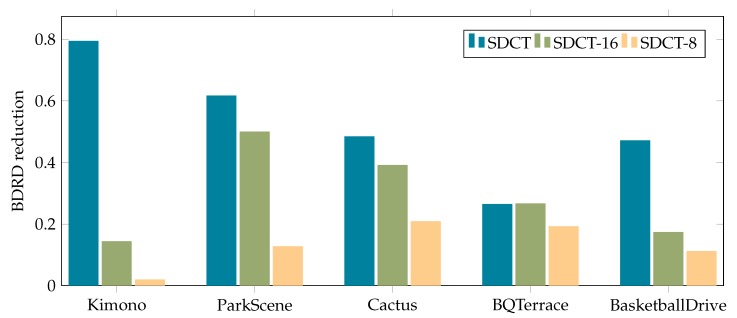
Histogram of obtained BDBR saving with respect to DCT.

Kimono, ParkScene, Cactus, BQTerrace and BasketballDrive are standard sequences employed to assess the encoder performances. The BDBR has been measured and the compression results are presented in Table 6 and Figure 8. As expected, the full SDCT presents a BDBR reduction but with a high computational cost. Reduced SDCTs are still able to maintain an average reduction, superior with respect to plain DCT compression. All the sequences have been compressed as *all intra* with default settings with Constant QP (*Quantization Parameter*) of values 22, 27, 32 and 37 for BDRD computation. Even when using only small SDCT transforms, the quality of the output is still better than the plain DCT. This is to be expected as the DCT can be seen as a special case of SDCT with steering angles of integer multiples of $\frac{\pi }{4}$.

### 4.2. Comparison with Previous Works

Since the Steerable-DCT is a new approach, it is not easy to make a fair comparison with other architectures found in the literature. However, for the sake of completeness, Table 7 proposes a comparison between the proposed SDCT architecture and some state of the art DCT ones. Zhao et al. [16] proposed an architecture able to support transform sizes from 4 × 4 to 32 × 32 with an implementation policy that reuses structure parts in order to contain the final dimension. Moreover, multiplications are substituted by shift and sum operations. Even though it uses a smaller technology compared to SDCT ( 45 nm vs. 65 nm) that grants a faster clock frequency (1.7×), the SDCT presents 4.7× higher throughput. Ahmed et al. [17] designed a folded structure that decomposes the DCT matrices into sparse submatrices to reduce the multiplications. Moreover, these last are eliminated thanks to a lifting scheme. Albeit such scheme supports 1080P HD video codec, its throughput is more than 12 times lower than the SDCT as well as the worst of those presented in Table 7 in terms of samples per second. Meher et al. [13] describe two versions of a pruned design: folded and full-parallel. Both present a working frequency equal to the SDCT, however, while the folded has also the same throughput, the full-parallel outperforms the rest since the hardware is replicated many times. Despite the SDCT follows a low-power paradigm with its folded-based structure, the hardware overhead needed to decompose the 2D-DCT transform results in superior power consumption. As a consequence, the pruned approach used in [13] grants a higher energy efficiency. Finally, Masera et al. [18] outline a folded approximated architecture with a just 7% higher throughput than SDCT, but with an energy per sample (EPS) comparable to the SDCT Folded-8 version.

## 5. Conclusions

The most recent state-of-the-art compression technique is the HEVC, which almost doubles the performance in terms of rate-distortion compared to the H.264/AVC. Nevertheless, the continuous development of new High Definition (HD) or Ultra-HD (UHD) techniques introduces high requirements concerning the storing and the transmission of such sequences of frames. Thus, researchers and companies are trying to push further the HEVC boundaries.

This paper provides an efficient and compact hardware architecture accelerator for the SDCT algorithm to be used in the HEVC algorithm. Many of the design choices explained above present an optimized approach, such as the lifting-based approach, in which the hardware resources are reduced to a minimum. Moreover, the flexibility showed by this architecture makes it appealing for a wide range of applications, being able to work with different coding formats. The proposed SDCT framework is able to cope with 8k UltraHigh Definition (UHD) (7680×4320 pixels) with a frame rate of 60 Hz for the 4:2:0 YUV format, which is one of the highest resolution supported by HEVC. The steerable DCT is a viable solution to improve compression efficiency, as reported in [8]. Further work will cover the integration of the proposed accelerator in a complete HEVC framework to validate the performances in a real case scenario.

## Figures and Tables

**Table 1:** Steerable Control Unit FSM states.

write input buffer	A	START
read input & write output buffer	B	WAIT
write input & read output buffer	C, F, I, L	WB
read input & write output & read output buffer	D, G, H, M	RWB
read output buffer	$E,E_{1},E_{2},E_{3}$	RB

**Table 2:** Example of FSM state evolution.

Memory Operation	Number of Cycles											
Write	16	X	16	X	8	X	4	X	4	12	X	X
Read & Write	X	16	X	16	X	8	X	4	X	X	16	X
Read	X	X	16	X	8	8	4	4	4	X	X	16
State	A	B	C	B	C	D	C	D	C	A	B	E
			16		16		8		4			16

**Table 3:** Estimated power consumption at 188 MHz.

Power	Internal	Switching	Total Dynamic	Leakage
basic DCT	36.55 mW	17.72 mW	54.47 mW	33μW
clock gated DCT	21 mW	12.52 mW	33.52 mW	30μW
basic SDCT	290.47 mW	60.33 mW	350.88 mW	106μW
clock gated SDCT	88.71 mW	59.85 mW	148.67 mW	94μW
clock gated SDCT-16	27.86 mW	28.97 mW	56.85 mW	27μW
clock gated SDCT-8	6.56 mW	7.20 mW	14.17 mW	7μW

**Table 4:** SDCT area occupation for different clock regimes.

Cell	1× Total Area	2× Total Area	4× Total Area	8× Total Area
SDCT	4,337,744μm${}^{2}$	3,042,226μm${}^{2}$	1,608,759μm${}^{2}$	1,301,522μm${}^{2}$
2D-DCT	438,866μm${}^{2}$	601,970μm${}^{2}$	455,150μm${}^{2}$	474,167μm${}^{2}$
IM	1,401,523μm${}^{2}$	820,032μm${}^{2}$	495,856μm${}^{2}$	335,932μm${}^{2}$
OM	2,377,837μm${}^{2}$	1,418,162μm${}^{2}$	482,048μm${}^{2}$	319,037μm${}^{2}$
FIFO	86,542μm${}^{2}$	110,594μm${}^{2}$	113,008μm${}^{2}$	110,604μm${}^{2}$
ROM	5895 μm${}^{2}$	22,228μm${}^{2}$	13,227μm${}^{2}$	33,223μm${}^{2}$

**Table 5:** Overview of the obtained architectures.

Architecture	DCT	SDCT	SDCT-16	SDCT-8
Technology (nm)	65	65	65	65
Frequency (MHz)	188	188	188	188
Power (mW)	33.52	148.67	56.85	14.17
Throughput	2.992 G	2.992 G	1.496 G	0.748 G
Area (mm${}^{2}$)	0.321	1.427	0.444	0.110

**Table 6:** BDBR [%] for implemented reduced SDCT sizes versus DCT-only.

Sequence	SDCT [8]	SDCT-16	SDCT-8
Kimono	−0.795	−0.144	−0.020
ParkScene	−0.617	−0.500	−0.128
Cactus	−0.485	−0.392	−0.209
BQTerrace	−0.265	−0.267	−0.193
BasketballDrive	−0.199	−0.174	−0.112
**Average**	−0.472	−0.295	−0.132

**Table 7:** Comparison of 2D-DCT and SDCT Architectures.

Design		Technology	Frequency	Throughput	Power	EPS
		[nm]	[MHz]	[Gsps]	[mW]	[pJ]
Zhao et al. [16]		45	333	0.634	-	-
Ahmed et al. [17]		90	150	0.246	-	-
Meher et al. [13]	Folded	90	187	2.992	40.04	13.38
	Full-parallel	90	187	5.984	67.57	11.29
Masera et al. [18]	Architecture 1	90	250	3.212	51.72	16.10
SDCT	Folded	65	188	2.992	148.67	49.69
	Folded-16	65	188	1.496	56.85	38
	Folded-8	65	188	0.748	14.17	18.94
